# Development and psychometric properties of the Stressors in Breast Cancer Scale

**DOI:** 10.3389/fpsyg.2023.1102169

**Published:** 2023-03-28

**Authors:** M. Victoria Cerezo, Lorena M. Soria-Reyes, Bella Pajares, Jaime Gómez-Millán, María J. Blanca

**Affiliations:** ^1^Department of Psychobiology and Methodology of Behavioural Sciences, University of Malaga, Málaga, Spain; ^2^Instituto de Investigaciones Biomédicas de Málaga, University of Malaga, Málaga, Spain

**Keywords:** breast cancer, stressors, breast cancer-specific assessment tool, healthcare strains, concerns about the future

## Abstract

**Background:**

A diagnosis of breast cancer generates psychological stress, due not only to treatment and its side effects but also to the impact on different areas of the patient’s daily life. Although there are instruments for measuring psychological stress in the cancer context, there is currently no tool for assessing stressors specific to breast cancer.

**Aims:**

The aim of this study was to develop the Stressors in Breast Cancer Scale (SBCS).

**Method:**

A panel of experts evaluated the clarity and relevance of scale items, providing validity evidence based on test content. Psychometric properties of the scale were then analyzed.

**Results:**

Validity evidence based on the internal structure of the SBCS was obtained through exploratory factor analysis (EFA) and confirmatory factor analysis (CFA), following a cross-validation strategy. The CFA supported a second-order factor model with five dimensions: physical appearance and sex strains, health and daily difficulties, interpersonal relationship strains, healthcare strains, and worries and concerns about the future. This structure was invariant across two groups distinguished by time from cancer diagnosis (less than 3 and 3 years or more from diagnosis). Reliability, based on McDonald’s omega and Cronbach’s alpha coefficients, ranged from 0.83 to 0.89 for factor scores, and reached 0.95 for total scores. Validity evidence was also provided by correlations with depression, anxiety, perceived stress, and perceived health and quality of life.

**Discussion:**

The results support the use of the SBCS for measuring stress as a stimulus in the breast cancer context. Implications for clinical practice and research are discussed.

## Introduction

The diagnosis of cancer generates psychological stress related not only to treatment and its side effects but also to its impact on the person’s daily life and social and family roles (Brocken et al., 2012). Breast cancer is no exception in this regard (Dooley et al., 2017; Tschuschke et al., 2017; Borgi et al., 2020). Stress in people with cancer has been linked to high levels of anxiety, depression (Burgess et al., 2005; Mehnert and Koch, 2008; Alagizy et al., 2020; O’Hea et al., 2020), sleep problems (Schell et al., 2019; De la Torre-Luque et al., 2020), lower self-care (Abdollahi et al., 2020), and low levels of satisfaction with life (Cerezo et al., 2020, 2022). Research in this context has also found that emotional distress is associated with poor adherence to treatment (Ochoa and Casellas-Grau, 2017), lower quality of life (Zhao et al., 2020), and even a reduced likelihood of survival (Cardenal et al., 2012). These relationships demonstrate the importance of identifying levels of stress and emotional distress among individuals with cancer.

According to Lazarus and Folkman (1984), psychological stress is a particular relationship between the person and the environment that is appraised by the person as taxing or exceeding his or her resources and endangering his or her well-being. Generally speaking, stress is activated when a situation is perceived as being out of control, unpredictable, and overloaded (Cohen et al., 1983), leading to physical, psychological, and social discomfort, as well as to changes at the brain, endocrine, and mental levels (Lazarus and Folkman, 1984).

Stress is defined both as a response and a stimulus (Cox and Griffiths, 2010; Biggs et al., 2017). Stress as a response refers to the body’s neurobiological, physiological, emotional or behavioral signals that are triggered during exposure to stressors (Selye, 1978; Espejo et al., 2011; Koh, 2018). Stress as a stimulus refers to environmental situations, external circumstances or events which produce these stress signals (Koh, 2018). Life events, chronic strain, and daily hassles are examples of stressors (Gaol, 2016; Koh, 2018).

The level of perceived stress is usually assessed with self-report measures. Numerous stress questionnaires have been developed (see University of California and National Institute on Aging, 2018), and they generally include both stressors and stress responses. One of the most popular instruments is the Perceived Stress Scale (PSS; Cohen et al., 1983), whose psychometric properties have been reported in general populations (Bastianon et al., 2020; Ruisoto et al., 2020; Chen et al., 2021; Figalová and Charvát, 2021) and in people diagnosed with multiple sclerosis (Wu and Amtmann, 2013), diabetes (Gillani et al., 2011), and heart problems (Leung et al., 2010). Another widely used instrument that includes a stress subscale is the Depression, Anxiety, and Stress Scales (DASS-21; Lovibond and Lovibond, 1995), whose psychometric properties have also been examined in general populations (Lee et al., 2019; Zanon et al., 2020) and in specific populations such as adolescents (Moore et al., 2016; Shaw et al., 2017), Chinese hospital workers (Jiang et al., 2020), the Korean working population (Jun et al., 2018), menopausal Arab women (Bener et al., 2016), and psychiatric patients (Ali et al., 2021). Both these scales are global measures of perceived stress that have also been used in people with cancer (Golden-Kreutz et al., 2004; Bener et al., 2016; Fox et al., 2018; Kumar et al., 2019; Cerezo et al., 2022; Soria-Reyes et al., 2023). However, for individuals who are facing particularly overwhelming experiences, such as a diagnosis of breast cancer, global measures of this kind should be complemented by other more specific ones.

To the best of our knowledge, two measures of perceived stress have been developed specifically for the cancer context: the Questionnaire on Stress in Cancer Patients (QSC-R23; Herschbach et al., 2003), and the Newly Diagnosed Breast Cancer Stress Scale (NDBCSS; Lee et al., 2013, 2021). The QSC-R23 is a 23-item scale that assesses daily stressors associated with cancer, and it evaluates five dimensions: psychosomatic complaints, anxiety, information deficit, daily restrictions, and social stress. However, this questionnaire applies to all cancer patients and does not address breast cancer-specific situations (e.g., physical appearance related to breast). The NDBCSS comprises 17 items evaluating four factors, labeled negative perception, threat, unpredictable, and facing challenge, and it is designed to measure stress perceptions in women newly diagnosed with breast cancer. However, this questionnaire includes items related both to stressors (e.g., loss of the breast will affect my life) and responses (e.g., I often cry), without differentiating between the two. In addition, and as it name indicates, it is focused on the beginning of the cancer process and hence does not explore the stress that individuals may experience at different stages of this process, including survival following successful treatment. Therefore, there is currently no instrument that measures stressors specific to breast cancer and which is applicable throughout the disease process.

The aim of this study was to develop and examine the psychometric properties of the Stressors in Breast Cancer Scale (SBCS), a tool designed to measure the factors that generate stress in women diagnosed with this condition, independently of the time from diagnosis. A validated instrument of this kind is essential for assessing the specific stressors that affect women with breast cancer, and it could help to guide psychological intervention. In a first step, we conducted focus groups with women with breast cancer (in the presence of psycho-oncologists) to identify the dimensions that would need to be assessed by the scale. We then drew up a battery of items covering the five dimensions identified: physical appearance and sex strains, health and daily difficulties, interpersonal relationship strains, healthcare strains, and worries and concerns about the future. A panel of experts then assessed the clarity and relevance of these items in order to select those for inclusion in the SBCS and to provide validity evidence based on test content. In a second step, the psychometric properties of the SBCS were analyzed in a sample of women diagnosed with breast cancer. This involved exploratory and confirmatory factor analysis to obtain validity evidence based on the internal structure, measurement invariance as a function of time from cancer diagnosis, item analysis, reliability of test scores, and validity evidence based on relationships with other variables, specifically depression, anxiety, general perceived stress, and perceived health and quality of life.

## Step 1: Design of the questionnaire and content validity

### Design of the questionnaire

To identify the dimensions to be assessed by the scale, we conducted two focus groups with women diagnosed with breast cancer. Each group comprised five women diagnosed with breast cancer and recruited through cancer care associations, and two psycho-oncologists with over 5 years professional experience in the field. Of the five women in each group, two were in the early stages of the disease and still receiving treatment; two had been diagnosed less than 3 years previously and had completed treatment but continued with regular check-ups; and one was a survivor of the disease at more than 5 years post diagnosis. These women, who ranged in age from 30 to 71 years (*M* = 50.5; *SD* = 13.28), did not form part of the sample that completed the final instrument. Their task in the focus group was to describe potential daily stressors in different areas of their lives, covering all stages of the disease and its treatment. This process identified five dimensions of interest: physical appearance and sex strains, health and daily difficulties, interpersonal relationship strains, healthcare strains, and worries and concerns about the future.

We then drew up a number of items as indicators of each dimension. Each item was worded in the same direction so that higher scores would indicate a greater level of stress. We also took into account the guidelines for item wording regarding the importance of clarity, brevity, representativeness, comprehensibility, relevance, specificity, and simplicity (Muñiz and Fonseca-Pedrero, 2019). The dimension *physical appearance and sex strains* refers to stressors arising from the disease that affect the body’s appearance and the woman’s interest in sex; *health and daily difficulties* concerns stressors related to states of emotional and physical discomfort and those that affect daily routines or habits; *interpersonal relationship strains* refers to stressors linked to relationships with others, including family and friends; *healthcare strains* reflects stressors associated with the hospital or healthcare context, at both the structural and functional level, as well as with interpersonal relationships that are formed in that context; finally, the dimension *worries and concerns about the future* refers to thoughts or fears about what life may bring in the short or long term.

The initial questionnaire, the Stressors in Breast Cancer Scale (SBCS), consisted of 28 items, each rated on a 5-point Likert-type scale: 1 = not at all stressful or is irrelevant to me; 2 = a little stressful; 3 = moderately stressful; 4 = quite stressful; 5 = very stressful. This reflects the response format used in other stress questionnaires (e.g., Byrne et al., 2007; Lima et al., 2017; Blanca et al., 2020). The battery of items was submitted to a panel of ten experts, who assessed the clarity of each item and its relevance to the target domain. The panel comprised three men and seven women, aged between 33 and 64 years (*M* = 49.56; *SD* = 11.58). Three of the panel were cancer patients, three were psycho-oncologists, and four were healthcare professionals (two oncologists and two nurses); all professionals were specialized in breast cancer and had between 3 and 32 years of clinical experience (*M* = 14; *SD* = 9.23).

### Validity evidence based on test content

The analysis of validity evidence based on test content was focused on the clarity and relevance of the items. Clarity refers to the simplicity of understanding an item, whereas relevance is the extent to which each item on a test is pertinent to the targeted domain (Sireci and Faulkner-Bond, 2014). Each expert rated item clarity and its relevance to the target domain (Osterlind, 1989) on a 5-point Likert-type scale (from 1: low degree of clarity/relevance, to 5: high degree of clarity/relevance). The protocol they were given included a definition of each domain and instructions for rating the items, including an example rating. The experts received the protocol *via* e-mail. We recorded sociodemographic data for each expert (gender, age, area of expertise, and years of experience).

### Data analysis

We began by calculating the means for item clarity and relevance, with a score higher than 3 being considered as indicating a potentially adequate item. To assess agreement, we calculated Aiken’s *V* index (Aiken, 1980, 1985), which ranges from 0 (disagreement) to 1 (perfect agreement). A *V* index of 0.70 or higher was interpreted as indicating a satisfactory association between an item and its respective dimension (Charter, 2003). We then computed the 95% confidence interval for the *V* index of each item (Penfield and Giacobbi, 2004) and eliminated those where the lower limit was below the aforementioned cut-off of 0.70. Confidence intervals were calculated using the program created by Merino and Livia (2009).

### Results

Means for item clarity and relevance were above 4 in all cases, indicating that the experts considered them to be clearly worded and relevant. However, four items were eliminated because the cut-off value of 0.70 fell within the 95% confidence interval of the *V* index for relevance. These items were: “People asking me all the time how I’m doing,” *V* = 0.80 [0.65, 0.89]; “People giving me lots of advice,” *V* = 0.80 [0.65, 0.89]; “Feeling that family members are always keeping an eye on me,” *V* = 0.80 [0.65, 0.89]; and “Having to have lots of medical tests,” *V* = 0.82 [0.68, 0.91]. The remaining 24 items yielded a *V* index above 0.70 and the lower limit of the confidence interval was above this cut-off value. The results of this content validity analysis are shown in Table 1. These 24 items formed the final version of the SBCS, whose psychometric properties were examined in Step 2 of this study.

**Table 1:** Means for clarity and relevance, and values of the V index and its 95% confidence interval.

Items	Clarity					Relevance				
	*M*	*SD*	*V*	95%LL	CIUL	*M*	*SD*	*V*	95%LL	CIUL
Physical appearance and sex strains										
1. Experiencing changes in physical appearance	4.70	0.68	0.93	0.80	0.97	4.67	0.71	0.99	0.78	0.97
2. They remove my breast or it is deformed	4.70	0.68	0.93	0.80	0.97	5.00	0.00	1	0.91	1
3. Losing interest in sex	5.00	0.00	1	0.91	1	4.70	0.68	0.93	0.80	0.97
4. Feeling less attractive	4.90	0.32	0.98	0.87	0.99	4.90	0.32	0.98	0.87	0.99
Health and daily difficulties										
5. Feeling pain in general	5.00	0.00	1	0.91	1	4.80	0.63	0.95	0.83	0.99
6. Feeling distress in general	4.60	0.699	0.90	0.77	0.96	4.90	0.32	0.98	0.87	0.99
7. Feeling tired	5.00	0.00	1	0.91	1	4.90	0.32	0.98	0.87	0.99
8. Finding it hard to do things that require effort (e.g., housework, picking up heavy objects)	5.00	0.00	1	0.90	1	4.80	0.42	0.95	0.83	0.99
9. Finding it hard to engage with hobbies or leisure activities	5.00	0.00	1	0.91	1	5.00	0.00	1	0.91	1
Interpersonal relationship strains										
10. Being restricted in going out with friends	4.60	0.699	0.90	0.77	0.96	4.50	0.85	0.88	0.74	0.95
11. Feeling that some friends are not interested in me	4.60	0.97	0.90	0.77	0.96	4.40	1.35	0.85	0.71	0.93
12. Finding it hard to take care of people who depend on me (e.g., children, grandchildren, parents)	5.00	0.00	1	0.91	1	4.80	0.632	0.95	0.83	0.99
13. Having arguments with my close family members (e.g., partner, children, parents)	4.90	0.32	0.98	0.87	0.99	4.00	1.49	0.85	0.71	0.93
14. Feeling that family members do not pay attention to what I say or think	4.70	0.95	0.93	0.80	0.97	4.60	0.97	0.9	0.77	0.96
Healthcare strains										
15. Not being able to see the oncologist about a problem	4.90	0.32	0.98	0.87	0.99	4.70	0.68	0.93	0.80	0.97
16. Having lots of medical appointments	4.90	0.32	0.98	0.87	0.99	4.50	0.85	0.88	0.74	0.95
17. Not understanding what the doctors tell me	5.00	0.00	1	0.91	1	4.70	0.95	0.93	0.80	0.97
18. Healthcare staff not treating all my symptoms as important	5.00	0.00	1	0.91	1	4.80	0.42	0.95	0.83	0.99
19. Healthcare staff not offering solutions to all my symptoms	4.80	0.63	0.95	0.83	0.99	4.60	0.97	0.90	0.77	0.96
Worries and concerns about the future										
20. Thinking I’m going to have a relapse	5.00	0.00	1	0.91	1	5.00	0.00	1	0.91	1
21. Making plans for the future	4.80	0.42	0.95	0.83	0.99	4.80	0.42	0.95	0.83	0.99
22. Thinking about how the disease will affect my work	5.00	0.00	1	0.91	1	5.00	0.00	1	0.91	1
23. Thinking about how the disease will affect my family	4.80	0.63	0.95	0.83	0.99	5.00	0.00	1	0.91	1
24. Thinking I’m going to die	5.00	0.00	1	0.91	1	5.00	0.00	1	0.91	1

## Step 2: Psychometric properties of the SBCS

Having designed the SBCS and obtained validity evidence based on test content, we then examined its psychometric properties in a sample of women diagnosed with breast cancer. This involved exploratory and confirmatory factor analysis to obtain validity evidence based on the internal structure, measurement invariance as a function of time from cancer diagnosis, item analysis, reliability of test scores, and validity evidence based on relationships with other variables, specifically depression, anxiety, general perceived stress, and perceived health and quality of life.

### Method

#### Participants

Participants were 420 Spanish women diagnosed with breast cancer. They ranged in age from 23 to 76 years (*M* = 51.94, *SD* = 8.95), with a mean time since diagnosis of 5.32 years (*SD* = 6.03). The inclusion criteria were being of legal age, not having difficulties in understanding the psychological tests to be administered, a diagnosis of breast cancer, and signing informed consent. Participants were recruited through cancer care providers and associations that offer support to women who have undergone surgery for breast cancer. The sample characteristics are shown in Table 2.

**Table 2:** Sociodemographic and clinical characteristics of the sample (*N* = 420).

Variables	%
Spanish region	
North	39.5
South	60.5
Age (years)	
<50	39.5
≥50	60.5
Marital status	
Married	71.9
Single	13.8
Divorced	10.7
Widowed	3.6
Educational level	
Primary	9.3
Secondary/High School	47.6
University	43.1
Occupation	
Homemaker	14.8
Employed	31
Unemployed	9
Sick leave	26.2
Retired	19
Breast cancer stage	
0	6.2
I	21.9
II	46.4
III	21
IV	4.5
Time since diagnosis	
<3 years	49.3
≥3 years	50.7
Treatment received	
Surgery	92.6
Axillary lymphadenectomy	46.7
Chemotherapy	62.1
Radiotherapy	62.1
Endocrine therapy	38.3
Monoclonal antibody	15.5
Breast surgery performed	
None	7.6
Conserving	52.9
Mastectomy without reconstruction	20.2
Mastectomy with reconstruction	19.3
Cancer sequelae	
None	48.6
Lymphedema	23.8
Other	27.6
Medical check-up every:	
None	1.2
3 months	26.7
6 months	43.8
1 year or more	28.3

#### Instruments

*Stressors in Breast Cancer Scale* (SBCS), described above in Step 1. The SBCS comprises 24 items related to five theoretical dimensions (physical appearance and sex strains, health and daily difficulties, interpersonal relationship strains, healthcare strains, and worries and concerns about the future), each rated on a 5-point Likert-type scale (1 = not at all stressful or is irrelevant to me; 5 = very stressful).

*Depression, Anxiety, and Stress Scales* (DASS-21; Lovibond and Lovibond, 1995), in its Spanish version (Daza et al., 2002). This self-report instrument comprises 21 items rated on a 4-point Likert-type scale (0 = did not apply to me at all; 3 = applied to me very much, or most of the time). It consists of three scales or dimensions, each with seven items: depression (e.g., “I felt down-hearted and blue”), anxiety (e.g., “I felt scared without any good reason”), and stress (e.g., “I felt that I was rather touchy”). Higher scores are indicative of a higher level of the construct measured by each scale. The total score can also be computed, indicating the level of general affective distress (Daza et al., 2002). McDonald’s omega and Cronbach’s alpha coefficients in the present sample were 0.90 for depression, 0.84 for anxiety, 0.90 for stress, and 0.95 for total scores.

*Perceived helplessness subscale of the 10-item version of the Perceived Stress Scale* (PSS-10; Cohen et al., 1983; Cohen and Williamson, 1988), in its Spanish version (Remor, 2006) that has been validated in Spanish breast cancer patients (Soria-Reyes et al., 2023). This subscale comprises six items (e.g., “In the last month, how often have you felt nervous and stressed?”), each rated on a 5-point Likert-type scale (0 = never; 4 = very often), with higher scores reflecting high levels of perceived stress. McDonald’s omega and Cronbach’s alpha coefficients in the present sample were 0.90.

*Perceived health* was measured with a single item (Atroszko et al., 2015), “How would you describe your health at present?,” rated on a 10-point Likert-type scale (1 = bad; 10 = excellent). This single item serves as a screening tool for patients’ self-rated health status, which has been shown to be a powerful predictor of future health and use of health services (Jylhä, 2009).

*Perceived quality of life* was measured with a single item (Atroszko et al., 2015), “How would you describe your quality of life at present?,” rated on the same 10-point Likert-type scale (1 = bad; 10 = excellent). Research suggests that single-item measures of quality of life may be useful self-reported screening tools among people aging with disabilities (Siebens et al., 2015).

#### Procedure

The study was approved by the Ethics Committee of the University of Malaga and was conducted in accordance with the ethical standards of the Declaration of Helsinki. A convenience sampling strategy was used to recruit participants. Women with a diagnosis of breast cancer were sent an e-mail by staff of their respective care provider, inviting them to take part in the study. The e-mail included information about the study objectives and procedure, and a link to the questionnaires, which were hosted in the form of a secure online survey. It was made clear that participation was entirely voluntary and that data collection was anonymous. No incentives were given. Before completing the questionnaires, participants were required to sign (electronically) informed consent authorizing the use of the information they provided for research purposes. The completed survey could not be submitted electronically unless all questions had been answered, and hence there were no missing data.

#### Data analysis

We first computed descriptive statistics for each item, specifically the mean score, standard deviation, and skewness and kurtosis coefficients. In order to obtain validity evidence based on the internal structure of the scale, and as a preliminary analysis, the sample was randomly divided into two groups. With the first sample group (*N* = 219) we conducted an exploratory factor analysis (EFA), using the program FACTOR (Lorenzo-Seva and Ferrando, 2006) with the parallel analysis procedure (Timmerman and Lorenzo-Seva, 2011) and the unweighted least squares estimation method with polychoric correlation and promin rotation (Lorenzo-Seva, 1999). Next, a second-order factor model with five first-order factors (physical appearance and sex strains, health and daily difficulties, interpersonal relationship strains, healthcare strains, and worries and concerns about the future) was tested by confirmatory factor analysis (CFA) in the second sample group (*N* = 201). The analyses, which were carried out using the EQS 6.3 software package (Bentler, 2006), were based on the polychoric correlation matrix and used maximum likelihood and robust estimation methods. Mardia’s normalized coefficient was computed to assess multivariate normality, with a value above 5 indicating that data are non-normally distributed (Bentler, 2006; Byrne, 2006), and supporting the use of robust estimation. The following goodness-of-fit indices (Bentler, 2006) were considered: the Satorra-Bentler chi-square (S-B χ^2^), the comparative fit index (CFI), the non-normed fit index (NNFI), and the root mean square error of approximation (RMSEA). Values of the CFI and NNFI above or close to 0.95 and values of the RMSEA less than 0.06 indicate a good fit (Hu and Bentler, 1999), while values of the RMSEA between 0.06 and 0.08 represent a reasonable fit (Browne and Cudeck, 1993).

Having tested the internal structure of the SBCS with the two sample groups, we performed a CFA with the total sample, following the same procedure as described above. To provide further validity evidence based on the internal structure of the SBCS, we also tested measurement invariance for the second-order factor model across two groups as a function of time from cancer diagnosis. To this end, the sample was divided into two balanced groups: less than 3 years (*N* = 207) and 3 or more years from cancer diagnosis (*N* = 213). We began by determining the baseline model for each group separately (Byrne, 2008), and then tested configural invariance to establish whether the number of factors and factor-loading patterns were the same across groups. Next, we examined metric invariance to test equality constraints on first-order factor loading and equality constraints on first-and second-order factor loading across groups. The equality of constraints was considered to be tenable if the decrease in CFI in the most constrained model was less than or equal to 0.01 in relation to the configural model (Cheung and Rensvold, 2002; Byrne, 2008).

We proceeded using the total sample for subsequent analysis, computing corrected item-factor correlations and corrected item-total correlations. Values greater than 0.30 were considered as satisfactory (De Vaus, 2002). Reliability of test scores was estimated by computing McDonald’s omega and Cronbach’s alpha coefficients, once again for each factor and the total score on the SBCS. Coefficients above 0.70 are generally considered as satisfactory (Viladrich et al., 2017). Finally, the average variance extracted (AVE) was also computed, with values greater than 0.50 being considered as acceptable (Fornell and Larcker, 1981).

To obtain validity evidence based on relationships with other variables, we computed Pearson correlation coefficients between scores on the SBCS and scores on depression, anxiety, other measures of general perceived stress, and perceived health and quality of life. Following Cohen (1988), correlations around |0.10| were considered small, those close to |0.30| moderate, and those around |0.50| strong.

Finally, we computed descriptive statistics for scores on the five dimensions of the SBCS, and also for the total score as a global measure of stress. These analyses were performed using IBM SPSS v24.

### Results

Descriptive statistics for each item of the SBCS are shown in Table 3. The majority of items showed deviation from the normal distribution.

**Table 3:** Mean (M), standard deviation (SD), and skewness and kurtosis for items of the SBCS.

SBCS items	*M*	*SD*	Skewness	Kurtosis
1	2.94	1.39	0.01	−1.25
2	2.80	1.52	0.18	−1.42
3	2.84	1.35	0.10	−1.12
4	2.90	1.40	0.08	−1.23
5	3.18	1.33	−0.15	−1.11
6	2.90	1.46	0.08	−1.35
7	3.25	1.34	−0.19	−1.14
8	3.12	1.36	−0.07	−1.19
9	2.81	1.28	0.12	−1.00
10	2.39	1.30	0.52	−0.89
11	2.07	1.25	0.92	−0.28
12	2.78	1.46	0.19	−1.32
13	2.48	1.41	0.47	−1.13
14	2.04	1.28	0.99	−0.24
15	3.00	1.46	−0.04	−1.34
16	2.74	1.42	0.19	−1.25
17	2.46	1.42	0.49	−1.09
18	2.73	1.44	0.24	−1.28
19	2.92	1.46	0.02	−1.37
20	3.51	1.43	−0.45	−1.15
21	2.51	1.36	0.40	−1.06
22	2.64	1.45	0.36	−1.21
23	3.06	1.47	−0.05	−1.37
24	3.10	1.60	−0.09	−1.55

In the EFA with the first sample group the Kaiser-Meyer-Olkin (KMO) sampling adequacy index was 0.92, and Bartlett’s test of sphericity was statistically significant, *χ^2^*(276, *N* = 219) = 3589.2, *p* < 0.001. The factor loadings above 0.30 from the EFA of the SBCS are shown in Table 4. The analysis yielded five factors consistent with the theoretical structure proposed when developing the scale: physical appearance and sex strains (Factor 2), health and daily difficulties (Factor 1), interpersonal relationship strains (Factor 5), healthcare strains (Factor 3), and worries and concerns about the future (Factor 4). Only one item (item 10, “Being restricted in going out with friends”) showed a high factor loading on two factors, namely health and daily difficulties, and interpersonal relationship strains. According to our proposed scale design, this item should load on the latter factor. Therefore, in the second sample group we tested the second-order factor model using CFA with item 10 loading on this factor. This analysis yielded a Mardia’s coefficient of 22.99, indicating the need to use a robust estimation method. The goodness-of-fit indices obtained in the CFA indicated a good fit: CFI = 0.98, NNFI = 0.98, RMSEA = 0.064, CI [0.054, 0.073], with all factor loadings being statistically significant and above 0.50.

**Table 4:** Factor loadings (above 0.30) from the EFA of the SBCS (*N* = 219).

Items	F1	F2	F3	F4	F5
1		0.81			
2		0.67			
3		0.72			
4		0.99			
5	0.53				
6	0.61				
7	0.70				
8	0.78				
9	0.78				
10	0.63				0.52
11					0.40
12					0.42
13					0.52
14					0.77
15			0.66		
16			0.47		
17			0.76		
18			0.88		
19			0.98		
20		0.32		0.45	
21		0.32		0.45	
22				0.64	
23				0.95	
24				0.52	

F1, Health and daily difficulties; F2, Physical appearance and sex strains; F3, Healthcare strains; F4, Worries and concerns about the future; F5, Interpersonal relationship strains.

Having analyzed the internal structure of the SBCS with the two sample groups, we then tested the second-order factor model using the total sample. The results from the CFA are shown in Table 5. Mardia’s coefficient was equal to 46.13, and the model again showed satisfactory goodness-of-fit indices, with values of the CFI and NNFI above 0.95 and the RMSEA close to 0.06. Table 6 shows the factor loadings, all of which were statistically significant and above 0.50.

**Table 5:** Goodness-of-fit indices for the second-order factor model of the SBCS for the total sample (*N* = 420) and by time from diagnosis (<3 years: *N* = 207; ≥3 years: *N* = 213), and tests of measurement invariance across time from diagnosis.

Model	S-B *x^2^*	*df*	CFI	NNFI	RMSEA	Δ CFI
Total sample	707.97	247	0.986	0.985	0.067 [0.061, 0.072]	
< 3 years	449.85	247	0.987	0.986	0.063 [0.054, 0.072]	
≥ 3 years	510.76	247	0.984	0.982	0.071 [0.062, 0.079]	
Configural invariance	960.82	494	0.986	0.984	0.067 [0.061, 0.073]	
Equality constraints on first-order factor loading	991.51	513	0.985	0.984	0.067 [0.060, 0.073]	<0.001
Equality constraints on first-and second-order factor loading	995.32	518	0.985	0.985	0.066 [0.060, 0.072]	<0.001

S-B *χ*^2^, Satorra-Bentler chi-square; df, degrees of freedom; CFI, comparative fit index; NNFI, non-normed fit index; RMSEA, root mean square error of approximation with 90% confidence interval; ΔCFI, CFI configural invariance model – CFI more constrained model.

**Table 6:** Factor loadings, corrected item-factor and item-total correlations, McDonald’s omega and Cronbach’s alpha coefficients, and average variance extracted for factor scores (*N* = 420).

SBCS items	Factor loading	Second-order factor loading	Item-factor correlation	Item-total correlation	*ω*/*α*	AVE
Physical appearance and sex strains		0.75			0.83	0.63
1	0.79		0.67	0.60		
2	0.77		0.63	0.61		
3	0.74		0.62	0.52		
4	0.87		0.75	0.60		
Health and daily difficulties		0.87			0.89	0.68
5	0.82		0.73	0.69		
6	0.80		0.69	0.68		
7	0.86		0.77	0.69		
8	0.82		0.74	0.65		
9	0.82		0.73	0.70		
Interpersonal relationship strains		0.87			0.83	0.59
10	0.77		0.63	0.65		
11	0.68		0.55	0.56		
12	0.78		0.62	0.65		
13	0.80		0.69	0.62		
14	0.79		0.66	0.55		
Healthcare strains		0.85			0.89	0.69
15	0.80		0.75	0.69		
16	0.73		0.66	0.71		
17	0.79		0.72	0.64		
18	0.92		0.79	0.72		
19	0.92		0.78	0.70		
Worries and concerns about the future		0.87			0.87	0.65
20	0.88		0.73	0.70		
21	0.75		0.66	0.61		
22	0.71		0.62	0.62		
23	0.80		0.70	0.69		
24	0.88		0.73	0.69		

Regarding measurement invariance across two groups as a function of time from cancer diagnosis, the analysis yielded adequate goodness-of-fit indices across the groups with less than 3 years and 3 or more years from diagnosis. The configural model and the models with equivalence of the first-order and second-order factor loadings also provided adequate goodness-of-fit indices. In addition, the decrease in the CFI value from the configural model to the more constrained models did not exceed 0.001. Table 5 shows the results of the measurement invariance analysis.

Table 6 also displays the corrected item-factor correlations and corrected item-total correlations. All values were above 0.30. McDonald’s omega coefficients were satisfactory, with values greater than 0.70 for factor scores, and equal to 0.95 for the total score. The same values were obtained when computing Cronbach’s alpha coefficients. As Doval et al. (2023) point out, differences between the two coefficients are generally reflected from the third decimal place onwards if data are derived from a population reliability that is reasonable for practical purposes (close to 0.80). Finally, the AVE ranged from 0.59 (interpersonal relationship strains) to 0.69 (healthcare strains), and hence all values were above 0.50.

Validity evidence based on relationships with other variables was provided by correlations with depression, anxiety, other measures of general perceived stress, and perceived health and quality of life. Correlations are shown in Table 7. Overall, total scores on the SBCS correlated positively and strongly with scores on depression, anxiety, and perceived stress. Correlations between these variables and factor scores were moderate or strong, the strongest correlation being that with scores on worries and concerns about the future. Associations with self-rated health and quality of life were negative and moderate both for factor scores and the total score. In this case, the strongest correlations observed were with scores on health and daily difficulties.

**Table 7:** Correlations between SBCS factor and total scores and scores on depression, anxiety, stress, perceived helplessness, and perceived health and quality of life (*N* = 420).

Variables	Physical appearance and sex strains	Health and daily difficulties	Interpersonal relationship strains	Healthcare strains	Worries and concerns about the future	Total score SBCS
Depression (DASS-21)	0.37^***^	0.37^***^	0.38^***^	0.36^***^	0.44^***^	0.44^***^
Anxiety (DASS-21)	0.39^***^	0.47^***^	0.46^***^	0.49^***^	0.53^***^	0.54^***^
Stress (DASS-21)	0.31^***^	0.33^***^	0.40^***^	0.39^***^	0.49^***^	0.45^***^
Affective distress (DASS-21 total score)	0.39^***^	0.43^***^	0.45^***^	0.45^***^	0.53^***^	0.52^***^
Perceived helplessness (PSS-10)	0.46^***^	0.41^***^	0.43^***^	0.41^***^	0.48^***^	0.51^***^
Perceived health	−0.35^***^	−0.40^***^	−0.32^***^	−0.31^***^	−0.37^***^	−0.40^***^
Perceived quality of life	−0.40^***^	−0.43^***^	−0.37^***^	−0.37^***^	−0.44^***^	−0.46^***^

^***^p < 0.001.

Having obtained evidence regarding the psychometric properties of the SBCS, we then computed descriptive statistics for scores on the five factors, and also for total scores so as to obtain a global measure of stress. Factor scores were calculated both as the mean and the sum of the respective item scores. Table 8 shows descriptive statistics for each of the five dimensions (i.e., physical appearance strains, health and daily difficulties, interpersonal relationship strains, healthcare strains, and worries and concerns about the future) and for the SBCS as a whole.

**Table 8:** Means, standard deviations, and skewness and kurtosis for scores on the SBCS (its five dimensions and total, *N* = 420) calculated as the sum or the mean (5 points) of the respective items.

SBCS scores	Number of items	*M*	*M* (5 points)	SD (5 points)	Skewness	Kurtosis
Physical appearance and sex strains	4	11.48	2.87	1.16	0.11	−1.03
Health and daily difficulties	5	15.27	3.05	1.13	−0.04	−0.99
Interpersonal relationship strains	5	11.76	2.35	1.04	0.50	−0.71
Healthcare strains	5	13.84	2.77	1.21	0.13	−1.13
Worries and concerns about the future	5	14.82	2.96	1.18	0.03	−1.09
Total score SBCS	24	67.16	2.80	0.96	0.04	−0.85

## Discussion

The purpose of this study was to develop and examine the psychometric properties of the Stressors in Breast Cancer Scale (SBCS), a tool designed to measure the factors that generate stress in women diagnosed with this health condition, independently of the time from diagnosis. The study was carried out in two phases. The first involved focus groups with women with breast cancer to identify dimensions of interest, development of a battery of items relating to the five dimensions identified, and evaluation of these items by a panel of experts to obtain validity evidence based on test content. In the second phase, we performed an exhaustive analysis of the psychometric properties of the resulting scale in a sample of 420 women with breast cancer, including validity evidence based on the internal structure, measurement invariance as a function of time from cancer diagnosis, item and reliability analysis, and validity evidence based on relationships with other variables, specifically depression, anxiety, general perceived stress, and perceived health and quality of life.

Validity evidence based on test content was obtained by analyzing the clarity and relevance of items. A panel of experts comprising both cancer patients and oncology professionals rated these two aspects (clarity and relevance) for each of the 28 items in the initial battery. After assessing agreement among experts, four items were eliminated as they were not considered relevant. The remaining 24 items were considered to adequately represent the following five dimensions: physical appearance and sex strains, health and daily difficulties, interpersonal relationship strains, healthcare strains, and worries and concerns about the future.

Next, we conducted EFA and CFA to obtain validity evidence based on the internal structure of the 24-item SBCS, following a cross-validation strategy. These procedures, performed with two random samples, showed an internal structure of the SBCS that supported the proposed theoretical structure based on the aforementioned five dimensions. The CFA yielded good fit indices for the second-order factor model with both the reduced sample and the total sample. In addition, measurement invariance was found across two groups as a function of time from cancer diagnosis, specifically, less than 3 years and 3 or more years from diagnosis. This indicates that items are indicators of the same latent factors and that the unit of measurement of the underlying factors is the same for both groups. The second-factor order structure enables both a score for each dimension and a total score to be obtained. The total score on the SBCS could be a useful measure of the impact of stressors on women with breast cancer, complementing other measures of general perceived stress that are not specific to this health condition, for example, the PSS-10 or DASS-21. According to Herschbach et al. (2004), cancer-specific distress scales offer more precise insight into patients’ experience than do general or psychiatric questionnaires.

The first of the five factors that make up the SBCS *is physical appearance and sex strains,* comprising four items referring to stressors arising from the disease that affect the body’s appearance and the woman’s interest in sex. Scores on this dimension were among the highest in the present sample, indicating that strains related to physical appearance are relevant stressors for these women. This is consistent with previous research showing that the assessment of body image enables a better understanding of the stress experienced by women with breast cancer (Guedes et al., 2018), and that body image dissatisfaction may lead to emotional distress, mood disorders, and impaired intimate relationships (Sebri et al., 2021).

The second SBCS factor is *health and daily difficulties*, which includes five items describing stressors related to states of physical discomfort such as fatigue or pain. These symptoms have been shown to be significant stressors for patients with different types of cancer (van den Beuken-van Everdingen et al., 2016; Draeger et al., 2018), and they are not limited to the treatment period (Mokhatri-Hesari and Montazeri, 2020). This factor yielded the highest scores in the present sample, suggesting that these symptoms should receive special attention and be addressed by multidisciplinary teams (nurse, physician, physiotherapist, psychologist, etc.).

The third factor is *interpersonal relationship strains,* comprising five items referring to stressors that originate in the context of social relationships, including family and friends. Previous research has shown that strains in family relationships can affect the well-being of both the woman with breast cancer and her close relatives (Segrin et al., 2018), and also that social support may be a protective factor in helping these women cope with stress (Ozdemir and Arslan, 2018). In the present sample, this dimension yielded the lowest scores. Future research should analyze in more detail the relationship between interpersonal relationship strains and social support in the breast cancer context.

The fourth factor is *healthcare strains*, composed of five items referring to stressors that result from the hospital and healthcare setting. It is acknowledged that patients continue to have healthcare needs throughout the disease process (Cherif et al., 2020), and also that factors such as long waiting times and difficulties communicating with staff can lead to dissatisfaction with the care provided (Tremblay et al., 2015). The fact that cancer survivors may, at some point, experience mental health problems such as depression, anxiety, and sleeping difficulties underlines the importance of ongoing access to healthcare (Cherif et al., 2020; De la Torre-Luque et al., 2020; Aggeli et al., 2021).

The fifth and final factor is *worries and concerns about the future,* comprising five items referring to stressors that reflect thoughts or fears about what life may bring in the short or long term. Previous research has shown that people with cancer typically fear tumor recurrence or metastasis, and this often leads to psychological sequelae (Koch et al., 2014; Cupit-Link et al., 2018). Accordingly, in the present sample, this was the dimension that yielded the second highest scores, and it also included the highest scored item on the SBCS (“Thinking I’m going to have a relapse”).

As regards the item analysis, correlations between item scores and both the score on their corresponding factor and the total score indicated that all items showed satisfactory homogeneity, with values above 0.50. Reliability, estimated by McDonald’s omega and Cronbach’s alpha coefficients, ranged from 0.83 to 0.89 for scores on the five factors, while the coefficients for the total score were 0.95, all above the 0.70 threshold. These results indicate satisfactory reliability of SBCS scores. The AVE was also above the 0.50 threshold for all factor scores, indicating that the amount of variance that is captured by the construct is larger than the variance due to measurement error (Fornell and Larcker, 1981).

Validity evidence based on relationships with other variables was provided by correlations between SBCS scores and scores on depression, anxiety, stress, affective distress (all with the DASS-21), the perceived helplessness subscale of the PSS-10, and perceived health and quality of life (both using single items). Overall, total scores on the SBCS correlated positively and strongly with scores on depression, anxiety, and stress, in line with previous research (Alagizy et al., 2020; Borgi et al., 2020). These results show that the impact of stressors experienced by women with breast cancer is reflected in their level of perceived general stress and other indicators of mental health. In terms of factor scores, scores on worries and concerns about the future showed the strongest correlations with these variables, indicating that thoughts or fears about what life may bring in the short or long term are associated with higher levels of affective distress. These results are consistent with previous research which found that fear of disease progression was the greatest concern for cancer patients (Cupit-Link et al., 2018), especially women with breast cancer (Herschbach et al., 2004).

In the present sample, we also found that scores on self-rated health and quality of life were negatively correlated with both the total score and factor scores on the SBCS, which again is consistent with previous studies (Zhao et al., 2020). The strongest correlations were observed for health and daily difficulties and worries and concerns about the future, indicating that perceived difficulties in everyday life and fears about what life may bring in the short or long term are, for women with breast cancer, the most relevant stressors associated with the perception of lower levels of health and quality of life.

The present study has a number of limitations that need to be mentioned. First, the SBCS is a self-report scale whose scores may be affected by response bias. Second, our use of a convenience sample may limit the generalizability of results. Third, participants were women diagnosed with breast cancer from various locations in Spain, and hence further research is needed to analyze the psychometric properties of the SBCS with other populations, thereby potentially paving the way for its use in cross-cultural studies. Finally, given the cross-sectional nature of the present study, causal relationships between stress and other indicators of mental health cannot be established.

These limitations notwithstanding, the study also has important strengths. In contrast to measures such as the QSC-R23 (Herschbach et al., 2003) that are designed to assess daily stressors among cancer patients in general, the SBCS has been developed specifically for the breast cancer context and takes into account the particular features of this health condition. The main advantage of the SBCS is its brevity and its potential applicability throughout the breast cancer process, including survival following successful treatment; the latter sets it apart from measures such as the NDBCSS (Lee et al., 2013, 2021) that are focused on the beginning of the disease process. Another point to consider is that the SBCS is a measure of stress as a stimulus that provides not only a total score but also scores for specific areas, and it has been shown here that these scores are invariant across two groups as a function of time from cancer diagnosis (less than 3 years and 3 or more years from diagnosis). The scale can therefore be used as a complement to other measures of stress as a response or of general perceived stress so as to reach a better understanding of an individual’s stress levels. The relationships between SBCS scores and other indicators of mental health, such as depression and anxiety, as well as with perceived health and quality of life, support its potential utility in the clinical setting. Specifically, the information obtained through the SBCS could help psycho-oncology professionals to target interventions at specific areas that generate stress in women diagnosed with breast cancer, with the aim of improving their psychological well-being. It would therefore be of interest in future research to determine cut-off points for SBCS scores to guide professionals when undertaking psychological assessment. Finally, the SBCS could also be a useful tool in research aimed at investigating the impact of stressors on mental health and analyzing potential variables that mediate or moderate this relationship, thereby allowing the empirical testing of theoretical models of stress in the context of breast cancer.

## Data availability statement

The raw data supporting the conclusions of this article will be made available by the authors, without undue reservation.

## Ethics statement

The studies involving human participants were reviewed and approved by Ethics Committee of the University of Malaga (55-2017-H). The patients/participants provided their written informed consent to participate in this study.

## Author contributions

MC: conceptualization, investigation, methodology and writing the original draft, the reviews and editing. LS-R: conceptualization, investigation, writing the original draft, and reviews. BP and JG-M: investigation and writing reviews. MB: investigation, methodology, supervision and writing the original draft, writing the reviews and editing. All authors contributed to the article and approved the submitted version.

## Funding

This study was supported by Grupo de investigación consolidado CTS-110, Junta de Andalucía y I Plan Propio y Transferencia de la Universidad de Málaga (ayuda de Publicación en Abierto, C.1).

## Conflict of interest

The authors declare that the research was conducted in the absence of any commercial or financial relationships that could be construed as a potential conflict of interest.

## Publisher’s note

All claims expressed in this article are solely those of the authors and do not necessarily represent those of their affiliated organizations, or those of the publisher, the editors and the reviewers. Any product that may be evaluated in this article, or claim that may be made by its manufacturer, is not guaranteed or endorsed by the publisher.
