# Supplementary material for: Development and psychometric properties of the Stressors in Breast Cancer Scale
Source: Front Psychol. 2023 Mar 28;14:1102169. doi: 10.3389/fpsyg.2023.1102169 (PMC10086352; doi:10.3389/fpsyg.2023.1102169)
Supplement: Supplementary file 1 [file Table_1.DOCX]

**ESCALA DE ESTRESORES EN CÁNCER DE MAMA**

A continuación, encontrará algunas situaciones que le pueden suceder en el transcurso de la enfermedad. Por favor, indique en qué medida o grado estas situaciones le generan preocupación, inquietud o estrés **durante el** **último mes**, siendo:

1 = Nada estresante (o no es importante para mí o no me ha ocurrido)

5 = Muy estresante

Cuando termine, por favor, compruebe que no ha dejado ningún enunciado en blanco.

| Ítems | Nada estresante | Un poco estresante | Moderadamente estresante | Bastante estresante | Muy estresante |
| --- | --- | --- | --- | --- | --- |
| 1. Sufrir cambios en el aspecto físico | 1 | 2 | 3 | 4 | 5 |
| 1. Que me extirpen la mama o se me deforme | 1 | 2 | 3 | 4 | 5 |
| 1. Perder el interés por el sexo | 1 | 2 | 3 | 4 | 5 |
| 1. Sentirme menos atractiva | 1 | 2 | 3 | 4 | 5 |
| 1. Tener dolor en general | 1 | 2 | 3 | 4 | 5 |
| 1. Sentir malestar en general | 1 | 2 | 3 | 4 | 5 |
| 1. Sentir cansancio | 1 | 2 | 3 | 4 | 5 |
| 1. Tener dificultades para hacer actividades que requieran un esfuerzo (p. ej., tareas de casa, coger peso, ...) | 1 | 2 | 3 | 4 | 5 |
| 1. Tener dificultades para realizar aficiones o actividades de ocio | 1 | 2 | 3 | 4 | 5 |
| 1. Tener limitaciones para salir con amigos | 1 | 2 | 3 | 4 | 5 |
| 1. Sentir que algunos/as amigos/as no se interesan por mí | 1 | 2 | 3 | 4 | 5 |
| 1. Tener dificultades para atender personas que dependen de mí (p. ej., hijos/as, nietos/as, padres, ...) | 1 | 2 | 3 | 4 | 5 |
| 1. Tener discusiones con mis familiares cercanos (p. ej., pareja, hijos/as, padres, ...) | 1 | 2 | 3 | 4 | 5 |
| 1. Sentir que mis familiares no me hacen caso | 1 | 2 | 3 | 4 | 5 |
| 1. No poder acceder al oncólogo ante algún problema | 1 | 2 | 3 | 4 | 5 |
| 1. Tener que ir muchas veces al médico | 1 | 2 | 3 | 4 | 5 |
| 1. No entender las explicaciones médicas | 1 | 2 | 3 | 4 | 5 |
| 1. Que el sanitario no le dé importancia a todos mis síntomas | 1 | 2 | 3 | 4 | 5 |
| 1. Que el sanitario no me dé soluciones a mis síntomas | 1 | 2 | 3 | 4 | 5 |
| 1. Pensar que voy a tener una recaída | 1 | 2 | 3 | 4 | 5 |
| 1. Hacer planes de futuro | 1 | 2 | 3 | 4 | 5 |
| 1. Pensar en cómo afectará mi enfermedad a mi trabajo | 1 | 2 | 3 | 4 | 5 |
| 1. Pensar en cómo afectará mi enfermedad a mi familia | 1 | 2 | 3 | 4 | 5 |
| 1. Pensar que me voy a morir | 1 | 2 | 3 | 4 | 5 |
